# Developing health and wellbeing in prisons: an analysis of prison inspection reports in Scotland

**DOI:** 10.1186/s12913-021-06337-z

**Published:** 2021-04-07

**Authors:** James Woodall, Charlotte Freeman

**Affiliations:** School of Health and Community Studies, Leeds Beckett University, Leeds, UK

## Abstract

**Background:**

The Scottish Prison Service (SPS) has been long regarded for its progressive policy approach to health promotion in prison. It is one of the few countries with a strategic plan for health promotion implementation. Given the paucity of understanding in relation to the concept of a health promoting prison, this study assessed routinely collected prison inspection data to understand and distil learning in regard the practical implementation of health-promoting prisons.

**Methods:**

Her Majesty’s Chief Inspector of Prisons for Scotland (HMIPS) oversees the independent inspection of all prisons. This desk-based study analysed openly accessible inspection reports from a public repository. The sample was limited to inspection reports using the 2018 revised Standards to ensure comparability between reports. Eight unique inspection reports meeting this criterion were downloaded between January and October 2020. The prisons had their inspections undertaken between May 2018 and January 2020. Data from the reports which focused on ‘health and wellbeing’ were inductively coded using NVivo 12 to support thematic analysis.

**Results:**

Results are presented against the values and principles outlined in the SPS’ own framework for promoting health in prison. All of the institution reports contained evidence of fairness and justice in their prison and understandings of health inequalities were recognised by staff. There were also examples of mutual (peer) support between people in prison; good relationships between staff and prisoners; and strong health promotion leadership. Conversely, some environmental conditions hindered the development of health promotion – this included staffing shortages and some practices fostering health inequity. Even where a prison was reported as having health promotion activities in place these were focused on a narrow range of individual risk factors such as smoking cessation or substance misuse. Far less attention was paid to wider health determinants.

**Conclusions:**

Scotland has been at the forefront of attempts to embed a health promoting prison philosophy in their justice system. Inspection data focusing on ‘health and wellbeing’ were analysed, but the analysis suggests that more could be done to ensure a health promoting setting. The way prisons inspectors are assessing health and wellbeing in particular areas is very narrow, with the focus exclusively on healthcare without a wider appreciation of how other areas of prison life can impact.

## Background

Scotland has a total prison population of 8,000 people which has grown by one-fifth in the last two decades [1]. While the Scottish prison population is relatively small, the imprisonment rate is 149 per 100,000 of the national population which is comparable to England and Wales, Bulgaria and the Ukraine [1]. Evidence shows that people in Scottish prisons face considerable health challenges consistent with those seen in other Western European populations [2] – drug and alcohol use, poor mental health and smoking being just a few of these [3]. For some time, however, the Scottish Prison Service (SPS) has been recognised as adopting a progressive and innovative approach to health promotion in prisons and often seen as an exemplar of policy development [4, 5]. Their approach has been synonymous with compassion and with an excellent understanding of health inequalities and how these are manifest in prison populations [5]. The reasons for progressive outlook are multifaceted, but may relate to: a political climate within Scotland that places emphasis on social justice as a core feature – current policy and practice in Scotland, suggests a political position in relation to incarceration, which is sustained by the belief that prison provides a window of opportunity; a focus on collaboration and continuity of care between the prison system and community; a less fragmented health care system; and SPS embracing a care-orientated approach in its philosophy [6, 7]. Despite this, Scotland continue to be at a ‘cross-roads’ in relation to how they seek to address and deliver health interventions in the prison setting [6] with evidence lacking in informing policy and practice decisions [8] and calls for more carefully planned and evaluated health-promoting interventions in this environment [9].

The health promoting prison is a way of conceptualising a healthy prison environment and was initially put forward by the World Health Organisation during the mid-1990 s. It is an idea which germinated from the ‘healthy settings’ philosophy, originating from the Ottawa Charter [10]. It recognises that health is not only determined by individual action and behaviour change, but by creating supportive environments and conditions for health in places that individuals interact [11]. While there has been a considerable focus on healthy cities and health-promoting schools, less emphasis has been placed on prisons [11]. This has shifted, however, with recognition that promoting health within a prison context is important from a humanitarian, public health and economic perspective [12].

The Scottish Prison Service has embraced many features of the healthy settings approach. Their original strategic vision for the health promoting prison, devised at the start of the millennium [13], was based on core values, such as integrity, honesty and justice as well as principles such as empowerment, equity, partnership and sustainability. Their approach was aligned coherently with the original WHO rhetoric of a health-promoting prison concept [14] and resonated with a broader healthy settings philosophy [5]. Despite this, action plans resulting from the framework failed to match the rhetoric, with individual lifestyle issues dominating core actions [13]. A number of decades later, there are still calls for Scottish prisons to be more health-promoting [15]. Indeed, this initial inability to move from strategy rhetoric to practical action was conceded by the Scottish Prison Service who stated:

*“This framework is keen to drive a holistic approach to a healthy prison but the reality is that …. work is often topic based … we recognise that the approach taken could increase the risks around ‘silo thinking’ and be less conducive to supporting local working between and within agencies seeking to promote healthy lives.”* ([5], p.14).

A commitment to health promotion by several of the prisons in Scotland can be seen through the expressions of control over health, both mental and physical, made by those serving their sentence within them [9]. Some commentators highlight how the variation in monitoring and evaluation contribute to a lack of progress on this [16] and a landscape of unlearned lessons pertaining to health promotion in prisons has been observed [8]. Many factors may help to maintain this status quo. Researchers studying prisons face multiple barriers to access and the prison service itself must manage diminishing resources including staff numbers [17].

There is routine and ongoing evaluation of the promotion of health and wellbeing for the prisons in many countries – although this mainly focuses on people in prison and less on prisoners’ families and staff. These independent inspections cover safety and decency and, as inspections take place without prior warning, can give vital insights into how a prison is run [18]. That these reports can indirectly ‘regulate prison conditions’ ([19], p.532) has been claimed, however, there is a dearth of strong evidence that service delivery or outcome is improved [20].

The 15 prisons in Scotland are inspected by Her Majesty’s Chief Inspector of Prisons for Scotland (HMIPS). As an independent body, they then publish their findings in relation to the treatment of and conditions for prisoners against the nine HMIPS standards for inspecting and monitoring prisons [21]. The nine standards were revised in 2018 from those previously published in 2015. The HMIPS Standards and associated Quality Indicators now cover:

- Standard 1 Lawful and transparent use of custody.
- Standard 2 Decency.
- Standard 3 Personal safety.
- Standard 4 Effective, courteous and humane exercise of authority.
- Standard 5 Respect, autonomy and protection against mistreatment.
- Standard 6 Purposeful activity.
- Standard 7 Transitions from custody to life in the community.
- Standard 8 Organisational effectiveness.
- Standard 9 Health and wellbeing [21]..

The 17 Quality Indicators within Standard 9 reflects the extent to which the prison takes all reasonable steps to provide care in line with all relevant NHS standard guidelines and evidence-based treatments. Further to this, they also evidence the extent to which healthcare staff in prisons prevent harms and promote health and wellbeing. As such, this Standard is inspected by Healthcare Improvement Scotland [22].

While there are other organisations who oversee prison standards and conditions (e.g. the Scottish Public Services Ombudsman (SPSO)), the analysis of prison inspection reports is becoming a useful way of understanding how health and wellbeing is understood and delivered in prison contexts [8]. This paper aimed to analyse data from the most recent prison inspection reports in Scotland. Given the prominence of the SPS and their approach to tackling health and wellbeing, the specific objective was to distil the learning and evidence derived from the inspectors to support the development of health promotion in prison.

The analysis focused on eight of the 15 prisons. These eight prisons were inspected using the most recent inspection standards (the seven other prisons were excluded because they were inspected using the 2015 standards as discussed later). This paper is timely given the increased scrutiny of regulation and inspection in prisons, particularly in relation to health and wellbeing. There has been a renewed emphasis on the role of the inspectors and their role in contributing to improved prisoner health outcomes [23].

## Methodology

Documentary analysis is a research approach that systematically analyses organisational and institutional reports [24]. Prison researchers working toward a greater understanding of health promotion in prison have largely neglected documentary methods, opting instead to pursue empirical approaches [25–27]. While these studies have substantially advanced critical debate and dialogue and added rich insight, they often focus on a single, or small number of settings which makes transferability of findings challenging given the heterogeneity of most prisons.

The analysis of prison inspection reports is becoming a useful way of understanding how health and wellbeing is understood and delivered in prison contexts [8, 28] and used to effect in assessing mental health provision [29]. Prison inspection reports are publicly available documents and can offer unique and independent insight into the strategy and operation of prison establishments and moreover give understanding of the “cross-section of strengths and needs” in the estate ([29], p.3). Despite this, prison inspection reports have been criticised for being very narrow in their conceptualisation of health and wellbeing – focusing predominantly on disease prevention and screening activities which arguably supports the safe running of the institutions as a primary outcome, with genuine prisoner health and wellbeing as a secondary [8].

The HMIPS reports were accessed from the website hosting the public repository. As this repository contained the full archive of inspection reports undertaken by the Inspectorate the sample was limited to the full inspection reports conducted in prisons using the 2018 revised Standards to ensure comparability between reports. Eight unique inspection reports meeting this criterion were downloaded between January and October 2020 each covering a single prison, with no prison being inspected more than once during this period and, as a result of the coronavirus pandemic, the suspension of the ongoing independent prison monitoring and inspection in March 2020 [30]. The eight prisons had their inspections undertaken between May 2018 and January 2020.

To ensure the data and text most relevant to the aims of the analysis were collected the following Quality Indicators underpinning Standard 9 which were reported by the Inspectors were focused upon [21]:

- 9.1 An assessment of the individual’s immediate health and wellbeing is undertaken as part of the admission process to inform care planning.
- 9.2 The individual’s healthcare needs are assed and addressed throughout the individual’s stay in prison.
- 9.3 Health improvement, health prevention and health promotion information and activities are available for everyone.
- 9.4 All stakeholders demonstrate commitment to addressing the health inequalities of prisoners.

Analysis of the data were undertaken on NVivo 12 by two researchers who cross-checked and discussed any areas of discrepancy in interpretation or coding. The introduction of computer-assisted qualitative data analysis software (CAQDAS) – such as NVivo – has been a valuable tool in the process of data analysis for qualitative researchers for some time now. Though not devoid of critics, CAQDAS has arguably assisted researchers in managing large data sets and potentially improved analytical rigour and efficiency [31–33]. NVivo allowed data to be coded and retrieved relatively easily. It was also useful in annotating data and recording ideas, thoughts and hunches. Initially the data were coded inductively and this identified 18 open codes. To organise the codes into more coherent thematic categories, the values and principles outlined in the SPS’ own framework for promoting health [13] were used as a structure to organise the data. All codes were therefore aligned to the values (fairness and justice) and principles of the framework (equity; empowerment; partnership; sustainability) to aid further interpretation.

### Findings

This section reports analysis from eight male prison inspection reports. It is organised under themes which were informed by the principles and values of the SPS’ framework for promoting health. Illustrative quotations have been used to support thematic areas and to facilitate interpretation of the data. The prisons have not been identified in the reporting.

#### Fairness and justice

All the institutions contained evidence of fairness and justice in their prison inspection reports. For most of the institutions this was reflected in the healthcare staff demonstrating respect for the prisoners when undertaking routine interactions. Within the data prisoners reported that they were fully involved in aspects of their health care and several inspection reports detailed how confidentiality was prioritised by healthcare staff with screening carried out in a room that maintained the prisoner’s dignity:

*“Staff explained the health screening process to prisoners, made sure that they understood its purpose; actively encouraged and supported prisoners to be fully involved in their screening.”* (Prison 5).

The understanding of health inequalities by health care staff, and the challenges and barriers prisoners faced as a result of them, was described in all the inspection reports along with the high levels of professionalism and human rights-based approach to the provision of care. Several inspection reports described how equality, diversity and human rights training was part of mandatory and ongoing training for all staff working in the health centre. In practice this meant that staff demonstrated a human rights-based approach both inside and outside the health centre with a positive and non-judgemental approach:

*“Inspectors observed patient consultations at which inequalities, sensitive practices, and the principles of a human rights approach were clearly embedded. Inspectors also observed a number of nurse-patient interactions outside of clinics where these qualities were also witnessed.“* (Prison 1).

The majority of inspection reports described how staff were observed to adapt their approach to their patients as a result of their understanding of health inequalities, for example, providing information about the range of services available in different languages and formats as required. This included the use of easy formats using pictures and images, using Language Line and interpreters for those whose first language was not English, and Braille:

*“Translation services were available for patients when English was not their first language.”* (Prison 3).

Several reports described how the relationship with healthcare staff was described as a positive one by the prisoners who felt that they were encouraged to make informed choices about their health care.

Several inspection reports described adverse environmental conditions. This included the high population numbers in the prison and resultant demand for services, particularly in relation to drug use, and the state of disrepair of some buildings:

*“The general state of repair of the health centre and medical rooms in the halls was poor. The environment used for the delivery of healthcare in the prison was not fit-for-purpose. There were multiple areas of damage to the walls, flooring and paintwork. Similarly, the health promotion building had damaged ceiling tiles with exposed insulation.”* (Prison 2).

In some prisons, the lack of staff in post made it difficult to provide the full range of activities, including the provision of clinics:

*“In addition, several nursing posts were sitting vacant and recruiting to them was proving challenging. This reflects the national picture, with many prisons having difficulties recruiting to key clinical posts. As a result, the demand on existing staff to deliver a comprehensive range of services was almost at its ceiling.”* (Prison 5).

There were concerns in one report that the reliance on agency staff as a result of vacant posts may mean that the healthcare team was unable to provide the full skill mix to deliver safe care.

#### Equity

Assessment screening tools were used on admission to prisons to identify immediate health and wellbeing needs of people arriving in prison:

*“On arrival to Prison 2 the immediate health and wellbeing needs of all patients were assessed using a standardised assessment screening tool…If someone was found to be unfit to be in custody, arrangements were made to transfer them out to secondary care. Screening of opiate withdrawal was only carried out if a patient reported that they were actively using drugs.”* (Prison 2).

However, one report described how the standardised health screening only occurred for those prisoners arriving during the day and those arriving at night would not be assessed until the next day. There were other examples of institutions where health needs were identified at reception but then information was not shared or follow up:

*“Not all individuals with a long-term physical health condition were identified on arrival at the prison, and those that had been were not always followed up in line with current best practice, or, had appropriate care plans and accurate and detailed assessment documentation.”* (Prison 6).

The majority of inspection reports also acknowledged inequity in the provision of information to all prisoners. Despite the recognition of the impact of health inequalities and the adoption of a human rights approach, many of the institutions did not provide printed information in languages other than English or in a format suitable for those with literacy issues:

*“Different versions of referral forms were available and although the forms used simple language and some had pictures, they were not available in different languages or suitable for patients with literacy issues.”* (Prison 4).

Though the forms used by the health care team had simple language and pictures, inspectors felt they were not suitable and, in several institutions, individuals were not asked if they had any issues with literacy. As a result, some individuals may not fully understand the information given about access to healthcare.

Similarly those who may have had issues reading or writing in English were not always able to maintain confidentiality around their health needs. Some reports gave examples of prisoners needing other people to translate information or complete referral forms:

*“The referral forms were not suitable for those with literacy difficulties or difficulty reading and writing in English. In these instances inspectors were told that other prisoners could be asked to complete the form for the prisoner. These processes breach patient confidentiality.”* (Prison 7).

In regards to equity, individuals with physical disabilities were not always placed in accessible cells, and several reports described the impact of institutions which were not designed to house people with disabilities such as the lack of wheelchair accessibility to cells and accessible toilets and showers:

*“There was only one accessible cell available within the prison. On discussion with [Institution] senior management and healthcare staff, inspectors were told that this cell had been identified as not fit-for-purpose as it did not have appropriate adaptations.”* (Prison 3).

### Empowerment

Within Standard 9 of the inspection reports, there was no mention of empowerment in its fullest sense. Instead referring to attributes more closely associated with psychological (or individual) empowerment, such as increased self-esteem or knowledge (i.e. access to information). All the inspection reports contained reference to health promotion within the prison. In the majority of reports, however, this reflected the provision of information about services available to prisoners:

*“Prisoners were encouraged and supported to take up the wide range of health promotion activities and opportunities available to them including; harm reduction, smoking cessation, sexual health, alcohol services and smart recovery groups.”* (Prison 4).

Some reports described how staff actively ‘encouraged and supported’ prisoners to take up the health promoting opportunities either through giving advice or through health promotion events for prisoners to both attend and raise awareness about. One example described the campaigns calendar used to focus on a different health issue each month.

Even where a prison was reported as having health promotion activities in place these were focused on a narrow range of individual risk factors such as smoking cessation or substance misuse. There was also an emphasis on transmissible disease through screening programmes such as those performing blood borne virus testing:

*“A range of national screening and immunisation programmes were available to prisoners including blood borne viruses (BBV), bowel screening and flu vaccinations. An opt-out model had been adopted for both the sexual health screening and the smoking cessation programmes. Staff told inspectors they would follow up with prisoners who had initially opted out of these at their admission, to check whether they wished to participate.”* (Prison 5).

There were examples of other areas where access to information and support was not widely available. Several institutions did not provide information about access to condoms for sexually active prisoners.

### Partnership

Evidence of collaborative partnerships were found in some prison inspection reports. These included individual institutions working in partnership with voluntary sector and community organisations to deliver health promotion activities or services for prisoners. One inspection report also described how the healthcare team had built relationships with community and voluntary sector groups to help support prisoners both prior to and after their liberation:

*“The Prisoner healthcare team had established strong working relationships with a range of third sector agencies, community groups and had processes in place to ensure that leading up to their liberation, patients were linked into the relevant community support prior to leaving prison.”* (Prison 2).

#### Mutual support

Peer support was described in some reports, often focusing on overcoming drug and alcohol problems. There were also examples of patient forums and focus groups in some institutions where issues relating to healthcare provision could be raised, though the levels of engagement were variable:

*“Despite efforts, prisoners in Prison 5refused to engage in the patient forums.”* (Prison 5).

All the prisons inspected showed evidence of their person-centred approach to the provision of care. Individuals could self-refer to the healthcare service where staff would discuss their concerns and signpost or refer to appropriate services. Those with complex needs often had anticipatory holistic care plans in place which were discussed and ‘owned’ by the patient and there was an emphasis on directly involving prisoners in their care:

*“Inspectors saw evidence that patients were being encouraged to be responsible for their own care, which was good practice.”* (Prison 8).

However, several reports demonstrated a lack of person-centred care within the institution:

*“Patients with a long-term condition were given limited support to self-manage their condition. Although care plans were in place, they were not person-centred or outcome-focussed and had not been developed in conjunction with the patient.”* (Prison 2).

In some institutions there was no evidence of a collaborative approach or reviews of ongoing care. One institution lacked a robust process for identifying those with long term conditions and *’relied on conditions being identified ‘‘opportunistically’ at other appointments’.*

### Sustainability

Health promotion leadership was described in some inspection reports, which manifested in highly motivated teams who were continually aiming to meet the needs of the prisoners in their care despite poor or challenging conditions. One report described the health promotion strategy for the prison:

*“The health promotion strategic lead had developed a health promotion strategy specifically for [Prison 5]. The strategy focuses on issues specific to both staff and prisoners and covers issues such as violence reduction, supporting the journey of recovery, smoke free prisons and staff health and wellbeing.”* (Prison 5).

There were good working relationships between healthcare and SPS staff as a result of close working between the senior management and an understanding and respect for each other’s critical roles in the wellbeing of prisoners. These positive working relationships led to good team dynamics and communication as well as an understanding of professional and ethical boundaries. This in turn led to positive impact on the health care of prisoners:

*“NHS and SPS staff within the health centre had a good understanding of each other’s roles and responsibilities and were seen to have a supportive working relationship. Effective two-way communication meant prisoners were supported to attend appointments within the health centre and within the community.”* (Prison 6)

However, in other institutions there was a lack of strong leadership with the health care staff feeling unsupported:

*“…many of the staff spoken with expressed feelings of vulnerability and of feeling unsupported in their roles by the healthcare leadership team.”* (Prison 8).

There were also reports of a lack of collaborative working with the wider prison staff team, including some reports of officers being abusive towards nursing staff, and the lack of resolution of longstanding operational issues such as the facilitation of attendance by prisoners at their healthcare appointments:

*“Inspectors were concerned that Prison 1] staff did not work together to solve problems which negatively impacted on the delivery of healthcare within the prison.”* (Prison 1).

One report described that processes to discuss operational issues were not in place amongst senior management.

## Discussion

This study sought to examine how health and wellbeing in the SPS is delivered according to independent prison inspection reports and to distil learning that could be used elsewhere to facilitate health-promoting prisons. This is particularly important given that health promotion in prison is poorly understood and variable in delivery within and across countries [34, 35]. It is clear that Scotland has been at the forefront of much of the attempts to embed a health promoting prison philosophy in their prison system [4]. They have adopted a progressive and forward-thinking ideology on health promotion which has been facilitated by a number of factors, including: the size of the SPS and the wider political position of the Scottish government [6, 7]. Their original framework for promoting health in prison [13] *“has been considered by those in the prison environment to have made a real difference”* ([5], p.6). While the rhetoric has been strong, there has, to date, been little critical examination of whether this is actually the case in practice.

While the findings and conclusions from this research come with methodological caveats (discussed later), it is apparent that there are many elements of the SPS’ approach to health and wellbeing could be replicated elsewhere and contribute more broadly to a greater understanding in how health promoting prisons are operationalised. The data suggests a clear understanding by staff of issues concerning health inequalities and an understanding of wider impacts of health. Staff were well-equipped to enable people in prison to access information and materials in different formats and moreover to provide opportunities for people to make informed choices. This included accessing health promotion events and programmes and the establishment of peer approaches to supporting people in prison with drug issues. Health promotion leadership seemed to be a factor which differentiated some of the prisons – where this was in place, it seemed to provide a stimulus for co-ordinated activity and seemed to foster positive relationships between all constituents of the prison, including SPS and NHS Scotland staff.

Despite positive findings, it is clear that when aspects of the reports are aligned to SPS’ own framework for promoting health the rhetoric seems to be in advance of the reality. Not all aspects of, for example, equity; empowerment; sustainability; mutual support, are being fully realised as outlined in strategic documents. While empowerment is raised as a key attribute of the health promoting prison in Scotland, it was clear that this focused on more individual notions and less about people in prison taking control of their wider factors impacting health. It was apparent that environmental conditions were not always ideal in terms of staff and physical resources. Indeed, some research has shown that health promotion initiatives are in short-supply with limited resource to satisfy everyone held within the institution [36]. When a prison did develop health promotion activities these often had a lifestyle focus which, it could be argued, was potentially futile in an environment where autonomous health decisions cannot be made. Issues concerning equitable provision were not universal across the prisons and issues such as confidentiality were not always taken seriously. Some of these barriers to operationalising a health promoting prison in Scotland have been recognised in stakeholder consultations, with environmental barriers and resourcing being an area which inhibits strategy being formulated into practice [37]. Variable leadership and commitment within the SPS institutions were also noted [5] and indeed re-emphasised in this study.

It has been argued before that inspection reports are overly negative and may fail to fully capture good practice in health service delivery [38] and this must be taken into full consideration. Also while the focus of Standard 9 was health and wellbeing, it was clear that this area of the report adopted a narrow conceptualisation of ‘health’ therefore the focus was almost exclusively on healthcare delivery. The SPS recognise that ‘health services’ are only one part of a wider team working in partnership with a prisoner, to help improve health [3]. This paper fully supports the point made by SPS on this matter:

*“the activities labelled as health promotion, the promotion of healthier lives continues, often unrecognised, within prisons. For example, the provision of healthy meal options, physical activity sessions, chaplaincy, work details, education, training and family visiting opportunities all contribute to both the physical and mental wellbeing of prisoners.”* ([5], p.10–11).

It could therefore be argued that features of the health promoting prison framework were seen elsewhere in other areas of the inspection, but not reported in Standard 9. This is possible, given other standards focusing on purposeful activity, for instance. However, it could be suggested that efforts relating to the health promoting prison would be reported, in the main, within Standard 9. Further research would be required to determine if that was the case, using the perspectives of prisoners as a primary methodological approach [5]. The paper suggests that the prison inspection methodology in Scotland for assessing health and wellbeing within Standard 9 currently offers a very narrow perspective. Indeed, we would argue that the criteria utilised reinforces health promotion as something that is reactionary and individualistic, addressing specific disease prevention targets that respond to the physical, psychological, emotional and social needs of individuals in only a partial way [39, 40]. This criticism is not unique and has also been reported in relation to narrowly defined inspection criteria in England and Wales [8].

The majority of concerted effort in relation to the health promoting prisons has been undertaken in the European WHO region, with Scotland leading the way in the debate and theoretical development [13]. There has been less political appetite for addressing the health of people in prison in other regions, such as North America [35] and Africa [41] and some of the lessons from Scotland could be transferred elsewhere. On a basic level, that could include health promotion leadership and a focus on how health information can be made accessible to all sectors of the prison population. It seems crucial to share good practice and policy approaches in relation to promoting health in prison – and examples from Scotland would clearly be part of this as evidenced in this study – but currently, dissemination of knowledge and experience beyond individual prisons, and indeed wider jurisdictions, is relatively uncommon. Such mechanisms for sharing policy and practice would seem critical to raise international standards in prison healthcare more generally.

As noted there are, of course, some limitations with the methodology employed in this research. The quantity and quality of information in the inspection reports was variable – a finding consistent elsewhere [29] – which does make it difficult to draw comparisons between institutions. In addition, these reports are not designed to be research data and therefore descriptions within them can be limited in depth [42]. Further drawbacks may be that inspectors may not report everything that they observe [42]. That said, this is the first assessment of health and wellbeing in Scottish prisons using routine prison inspection reports. It is hoped that future research will extend this analysis further.

## Conclusions

Scotland has been acknowledged as one of the countries which has taken a forward-looking and progressive view of health promotion in prisons. Theoretically and conceptually, the development of ideas in relation to health promotion in prison has emerged from Scotland (as well as England and Wales). Few, if any, nations have developed strategy to the same extent. An understanding of how this filters to delivery in practice is largely unknown with limited evidence or evaluation of this. This study sought to use prison inspection reports to assess Scottish prisons in relation to health and wellbeing. While there is much to admire and transfer in the way health promotion is operationalised in Scottish prisons – including an awareness of inequality and strong leadership in some institutions – it is apparent that this is not universal across the prison estate. The rhetoric of the health promotion in prison seems to be ahead of the reality of practice.

The paper argues that the way prisons inspectors are assessing health and wellbeing is potentially narrow. This study does have limitations which should be considered when drawing conclusions. For example, the research focused only on one section of the prison inspection (i.e. Standard 9 – Health and wellbeing) and therefore other areas of the report may have revealed more activities relating to those commensurate to a health promoting prison. This may be unlikely, however, given recent critique that a ‘whole-prison approach’ to health promotion was lacking in some countries [8, 23]. Notwithstanding the limitations of the current study, a prison inspection methodology focusing on health and wellbeing may be refined by reducing the focus on healthcare and appreciating how other areas of prison life may also impact (e.g. chaplaincy, family contact, gym, resettlement planning). This research has opened up the potential for further research to consolidate or contest the findings presented here. Further exploration, from the perspective of people in prison and staff, is needed to ascertain the extent to which prisons support or inhibit health and wellbeing.

## Data Availability

Reports are freely available here: https://www.prisonsinspectoratescotland.gov.uk/publications.
